# Natural Constraints to Species Diversification

**DOI:** 10.1371/journal.pbio.1002532

**Published:** 2016-08-09

**Authors:** Eric Lewitus, Hélène Morlon

**Affiliations:** Institut de Biologie, École Normale Supérieure, Paris, France; University of California, Berkeley, UNITED STATES

## Abstract

Identifying modes of species diversification is fundamental to our understanding of how biodiversity changes over evolutionary time. Diversification modes are captured in species phylogenies, but characterizing the landscape of diversification has been limited by the analytical tools available for directly comparing phylogenetic trees of groups of organisms. Here, we use a novel, non-parametric approach and 214 family-level phylogenies of vertebrates representing over 500 million years of evolution to identify major diversification modes, to characterize phylogenetic space, and to evaluate the bounds and central tendencies of species diversification. We identify five principal patterns of diversification to which all vertebrate families hold. These patterns, mapped onto multidimensional space, constitute a phylogenetic space with distinct properties. Firstly, phylogenetic space occupies only a portion of all possible tree space, showing family-level phylogenies to be constrained to a limited range of diversification patterns. Secondly, the geometry of phylogenetic space is delimited by quantifiable trade-offs in tree size and the heterogeneity and stem-to-tip distribution of branching events. These trade-offs are indicative of the instability of certain diversification patterns and effectively bound speciation rates (for successful clades) within upper and lower limits. Finally, both the constrained range and geometry of phylogenetic space are established by the differential effects of macroevolutionary processes on patterns of diversification. Given these properties, we show that the average path through phylogenetic space over evolutionary time traverses several diversification stages, each of which is defined by a different principal pattern of diversification and directed by a different macroevolutionary process. The identification of universal patterns and natural constraints to diversification provides a foundation for understanding the deep-time evolution of biodiversity.

## Abstract 2

A meta-analysis of 214 family-level vertebrate trees containing more than 12,000 species and spanning 500 million years of evolution reveals general patterns of species diversification and the constraints acting on them.

## Author Summary

Are there universal laws in the evolution of biodiversity? Why do some clades go extinct and others flourish? These questions are fundamental to our understanding of present-day biodiversity. In a meta-analysis of nearly 12,000 species spanning ~500 million years of evolution, we find that there are five principal patterns of diversification to which all vertebrate families hold, and that these patterns can be mapped into a multidimensional phylogenetic space. Importantly, because certain diversification patterns invariably lead to extinction, clades do not explore all possible phylogenetic space, and thus the evolution of biodiversity is constrained by a set of loose but inviolable rules. We characterize the biotic and abiotic factors precipitating those rules with important implications for our knowledge of the emergence and maintenance of the diversity of life around us.

## Introduction

The radiations and extinctions of species are recorded in the patterns of the tree of life [1,2]. Those patterns are fundamentally important to testing classical evolutionary hypotheses (e.g., the Red Queen [3] and adaptive radiation [4]), for understanding species distributions across clades [5] and regions [6], and, more generally, for piecing together how life evolves on Earth. Accordingly, the development of models for understanding how diversification unfolds using phylogenies of extant taxa has exploded in the last decade [7], and several meta-analyses have attempted to identify general principles of diversification [8–12].

Despite these recent and rapid developments, we do not yet have a coherent, panoramic view of the patterns of diversification across the tree of life. There is a general consensus that many phylogenies have a signal of slowdown in speciation rates through time, although this consensus is not universally accepted, and the processes behind the general pattern remain equivocal [8–13]. There is also broad agreement that diversification rates vary considerably across lineages [5,14], although approaches for detecting where diversification shifts happen [5,15] and what determines these shifts continue to be deliberated and debated [16]. Although model-based approaches have been crucial for testing specific hypotheses, a thorough description of the diversification patterns seen in empirical phylogenetic trees requires approaches free of any a priori assumption of how those trees behave.

We developed a novel, model-free framework rooted in spectral graph theory that allows for the direct comparison of phylogenetic trees across various groups of organisms (see Materials and Methods) [17]. In essence, we summarize the information contained in tree shape by a distribution—the spectral density—representing the spectra of eigenvalues of the modified graph Laplacian (MGL), which is a matrix constructed from the evolutionary distances between phylogenetic nodes specially designed to retain maximal information from the tree. This allows us to measure the similarities and dissimilarities between trees from different species clades by calculating the dissimilarity between their respective spectral densities. Phylogenetic trees can then be clustered according to how similar their shapes are. In addition, spectral density profiles can be used to compute summary statistics. In particular, the ln-transformed principal eigenvalue (λ*), skewness (*ψ*), and peak height (*η*) are indicative of phylogenetic expansion, stem-to-tip distribution of branching events, and heterogeneity of branch lengths, respectively [17]. Phylogenetic expansion λ* is highly correlated with measures of phylogenetic diversity and (less so) to species richness; the stem-to-tip distribution *ψ* is loosely correlated with the classical gamma summary statistic; and the heterogeneity of branch lengths as measured by *η* is not correlated with any known measure of phylogenetic balance but instead is representative of regular branching events in the tree (such that, for example, trees with localized radiations or diversification rate shifts will appear irregular). Importantly, trees constructed from different models of diversification cluster separately according to their spectral densities, and the three spectral density summary statistics are collectively sufficient to distinguish between trees governed by different diversification processes [17]. Thus, empirical trees can be grouped into clusters representing distinct diversification patterns and mapped with minimal information loss onto a three-dimensional space—which we call phylogenetic space—defined by λ*, *ψ*, and *η*.

## Results

**Fig 1:**
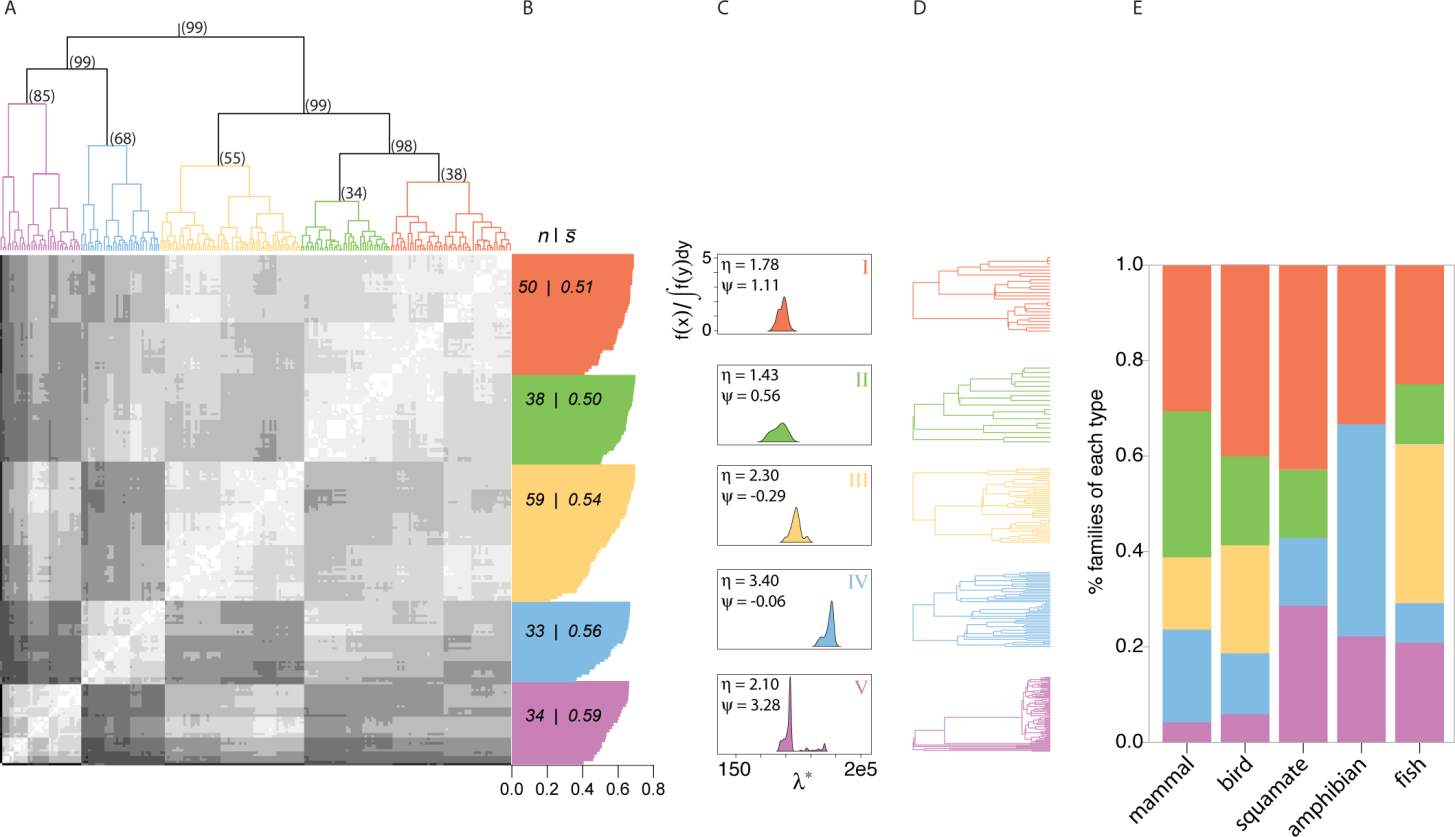
Five principal patterns of vertebrate diversification. (A) Heatmap and hierarchical clustering of spectral density profiles for 214 vertebrate phylogenies. Bootstrap probabilities are shown above each bifurcation. (B) Silhouette widths (*s*_*i*_) for each tree calculated from k-medoids clustering on spectral density profiles; the number of trees belonging to each cluster (n); and average silhouette width for each cluster ($\bar{s}$). (C) The median spectral density profile for each cluster (where the median profile is that which is closest to the median value for λ*, *ψ*, and *η*) and (D) apposite tree. Median values for *ψ* and *η* are listed in (C). (E) The percentage of each class of vertebrates belonging to each diversification cluster. The phylogenies included in the analyses are listed in S1 Data.

We computed the spectral density profiles of 214 family-level trees representing mammals [18], birds [19], squamates [20], amphibians [21], and ray-finned fishes [22] (see Materials and Methods), totaling 11,930 species. By clustering these profiles, we found that there are five principal ways in which all vertebrate families diversify, significantly supported by both hierarchical and k-medoids clustering (Fig 1A and 1B, respectively). Iterating our analyses on samples from a Bayesian posterior distribution of trees showed that these results were robust to phylogenetic uncertainty (Methods): we identified five clusters 100% of the time; in 75% of the iterations, the distribution of trees across clusters was identical; and in the iteration with maximum mismatch, only 6% of the trees were placed in different clusters. The five distinct clusters are characterized by both global-scale differences related to overall tree shape (as measured by *λ*, *η*, and *ψ*) and local-scale differences in branching patterns restricted to specific parts of the tree that are not encapsulated by the three main summary statistics but appear as smaller details in the shape of the spectral density profile (Fig 1C and 1D). Diversification types fall on a positively correlated gradient of λ* and *η*, with *ψ* varying considerably and unsystematically between types. The most abundant type (III) is defined by an intermediate λ* and *η* and is distinguished from the second most abundant type (I) by a relatively low *ψ*. The third and fourth most abundant (II and V) are virtually antipodal, the former having extremely low λ* and *η* and the latter being principally defined by a remarkably high *ψ*. The least abundant type (IV) is distinguished by very high λ* and *η*. Notably, as estimated by the eigengap heuristic, phylogenies were most frequently unimodal (40%), with 20% having two or three modes and the rest having between four and thirteen (S1 Data). No diversification type shows a prevalence for a number of modalities (D < 0.25, *p* > 0.1).

Despite variation in the abundance of types across families, we see no single diversification type that dominates the vertebrate landscape (Fig 1E, S2 Data). We observe, however, certain diversification types to be over- or under-represented within each vertebrate class, even when accounting for sample size (S1A Fig, S3 Data). Ray-finned fish deviate the least from an even distribution. Type III trees are absent in squamates and amphibians, but only significantly so in amphibians. This may be due, in part, to mean differences in crown age between classes (S1B Fig).

**Fig 2:**
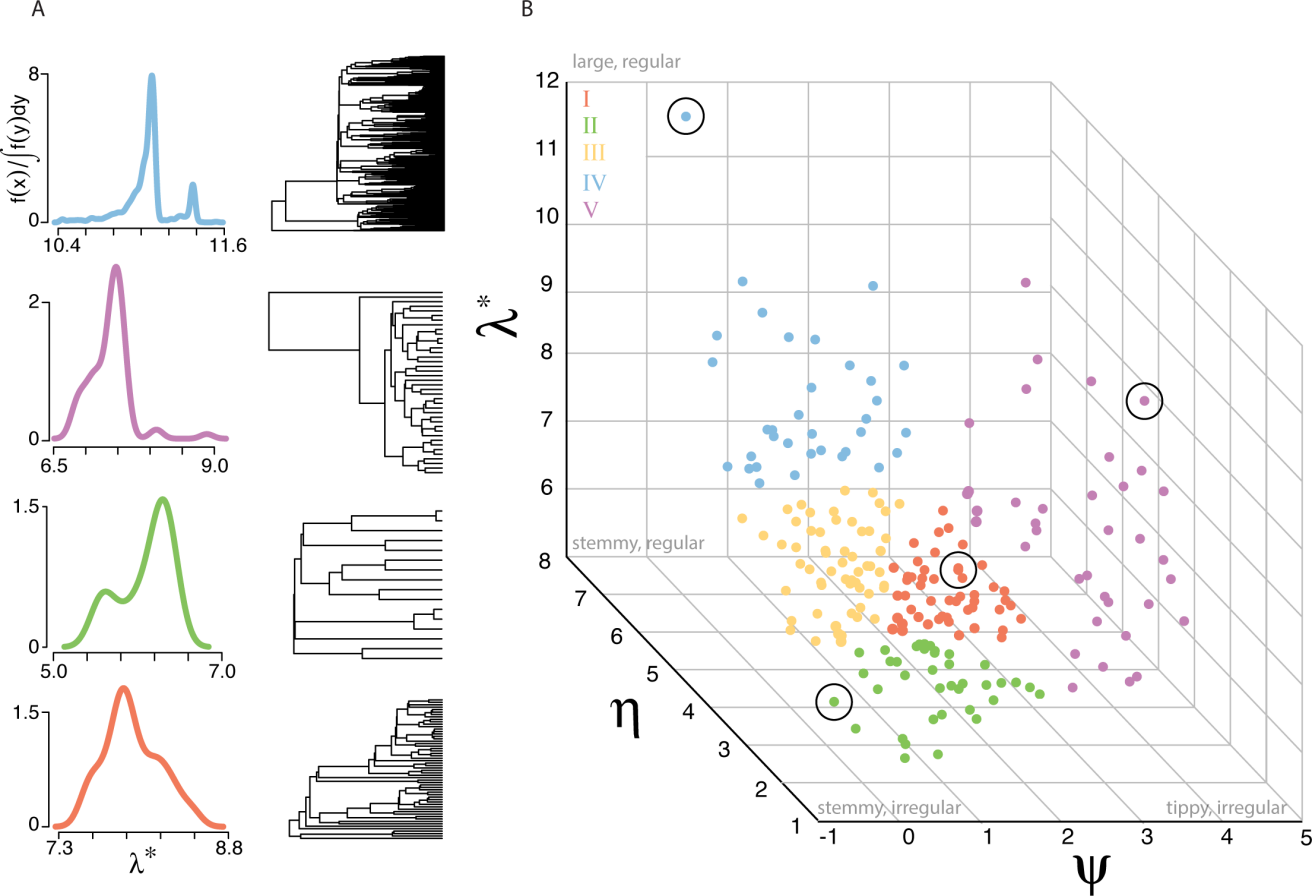
Vertebrate phylogenetic space. (A) Spectral density profiles and trees representative of different regions of phylogenetic space are shown: (top to bottom) Acanthizidae, Muridae, Pittidae, and Leporidae. (B) The distribution of trees in phylogenetic space defined by spectral density profile summary statistics: skewness (*ψ*), ln-transformed principal eigenvalue (λ*), and peak height (*η*). Points in phylogenetic space corresponding to trees in (A) are circled. Points in (B) are colored according to Fig 1. Summary statistics are listed in S1 Data.

Mapping empirical phylogenies in the space defined by λ*, *ψ*, and *η* reduces the high dimensionality of phylogenies to only three dimensions, thus allowing the first visualization of vertebrate phylogenetic space (Fig 2A). The geometry of phylogenetic space reveals that vertebrate phylogenies are able to occupy a broad, multidimensional range of tree space and therefore differ from one another along multiple axes of variation rather than fall along a single line. As expected, phylogenies from distinct diversification types cluster together in phylogenetic space and, accordingly, vertebrate classes are differently distributed in space (S1C Fig). Phylogenies differing in the number of missing extant taxa are spread across phylogenetic space, suggesting that undersampling does not bias our representation of this space (S2 Fig).

**Fig 3:**
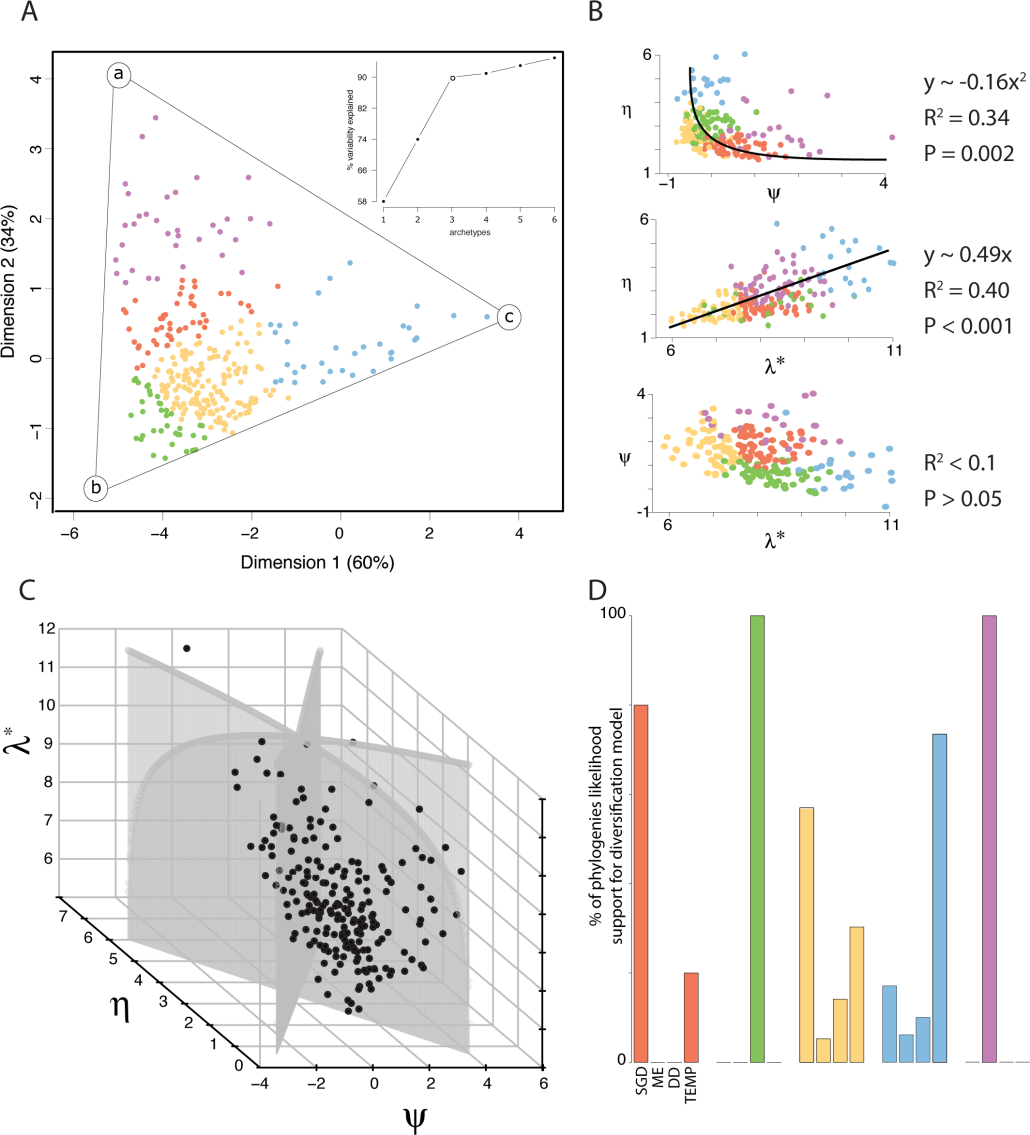
Constraints and trade-offs in vertebrate phylogenetic space. (A) The geometry of phylogenetic space (in Fig 2B) is best fit by a triangle, with each vertex corresponding to an archetype defind by a unique set of phylogenetic properties. (A, inset) The explained variability of the data distribution elbows at three vertices. (B) The trade-offs between phylogenetic properties in vertebrate phylogenetic space. (C) The set of empirical trees (black) and simulated phylogeny-type networks (grey) plotted in phylogenetic space. (D) Barplots of the percentage of empirical phylogenies of each diversification type with maximum likelihood support from a particular diversification model. Points in (A) are colored according to Fig 1. See S1 Data for summary statistic and model AICc values and S4 Data for model percentages.

The dimensionality of phylogenetic space can be further reduced to only two dimensions, as revealed by a polytope test that estimates the statistical significance of a given number of vertices (i.e., archetypes) encompassing input data (see Materials and Methods) [23]. We identified three archetypes as the minimum number that could significantly delimit phylogenetic space (*p* < 0.01). These three archetypes present a Pareto-optimal situation: all phylogenies fall within the optimal polytope (here, a triangle) and a different phylogenetic property is maximal nearest each archetype (i.e., vertex of the triangle) (Fig 2B). Each archetype is variably contributed to by λ*, *ψ*, and *η*—archetype *a* (10%, 89%, 1%); archetype *b* (44%, 6%, 49%); archetype *c* (45%, 4%, 51%)—such that, as a tree approaches an archetype, its properties change according to the proportional contributions of each property at that archetype. Direct trade-offs between λ*, *ψ*, and *η* reveal constraints on how certain phylogenetic properties covary (Fig 3A). The positive linear relationship between λ* and *η* reveals the tendency for phylogenies to become more regular as they accumulate phylogenetic diversity, with regularity increasing at a slower rate than diversity. The negative logarithmic describing *η* as a function of *ψ* draws a steep slope around *ψ* = 0, where a wide range of *η* is possible, but then abates, signifying the tendency for phylogenies (with *ψ* > 0) to become increasingly irregular as they become more tippy. Taken together, the archetypes underline phylogenetic trade-offs in the movement through phylogenetic space: as trees move from archetype *a* to *b*, they become more stemmy, and as they move from *b* to *c*, they become more expansive and regular.

The trade-offs we observe in phylogenetic space reveal biological rather than structural constraints. Comparing the three-dimensional empirical phylogenetic space to the entirety of the space available to phylogeny-type networks (i.e., bifurcating, ultrametric, and rooted trees restricted to the λ* range of vertebrate trees; see Materials and Methods) shows that empirical phylogenies occupy only a small portion (36%) of this possible space (Fig 3B). In fact, any combination of high, low, and medium values of λ*, *η*, and *ψ* can be reached by simulated phylogeny-type networks. The polytope encompassing phylogeny-type networks ranges more extensively in high-*η*, low-*ψ* phylogenetic space, resulting in a symmetrical, Pareto-poor geometry (S3 Fig). The difference between empirical and potential space is not linked to the restricted number of taxonomic families we analyzed in comparison with the total number of vertebrate families. Indeed, rarefaction analyses suggest that the sample of trees we analyze saturates empirical phylogenetic space, with coordinates of the two-dimensional polytope reaching an asymptote (S4 Fig). Hence, empirical phylogenetic space is truly evolutionarily constrained, with the parts of space epitomizing trees that are regular (high *η*) and either particularly stemmy (low *ψ*) or particularly tippy (high *ψ*) not reached by diversification trajectories. To understand what types of trees, in diversification terms, occupy these empty spaces, we simulated ultrametric trees under varying diversification parameters. We found that much of the regular space could be occupied by the simulated trees, with the stemmy space occupied by trees with a declining speciation rate and the tippy one occupied by trees with an increasing speciation rate (S5 Fig). There is overlap between the simulated and empirical trees, but most trees simulated with *β* = ±0.1 fall outside of vertebrate phylogenetic space.

In order to interpret different regions of phylogenetic space in traditional macroevolutionary terms, we estimated the statistical support for state-of-the-art, processed-based phylogenetic diversification models that are comparable in a likelihood framework (see Materials and Methods). The processes featured by these models were mass extinction events (constant speciation and extinction punctuated by events when a fraction of species is lost [24]), diversity dependence (diversification rates varying as a function of the number of extant species [25]), temperature dependence (diversification rates varying as a function of temperature [26]), and speciation by genetic differentiation (SGD) (speciation emerging from the accumulation of mutations [27]). Each diversification type is predominantly (or exclusively) supported by a different model. Specifically, 100% of type II phylogenies are best supported by an exponential diversity-dependent model; 100% of type V by a mass extinction model; 80% and 20% of type I by SGD and temperature dependence, respectively; phylogenies of type III and IV show support from all four diversification models, although they are disproportionately supported by SGD and temperature dependence, respectively (Fig 3C, S4 Data). Linear diversity dependence and linear temperature dependence were never selected above their exponential counterparts. On average, corrected Akaike Information Criterion differences (ΔAICc) between models showing the best and second-best likelihood support was modest (ΔAICc = 1.49±1.22; S6 Fig, S1 Data), which illustrates the well-recognized difficulty of unambiguously disentangling different diversification scenarios with model-based phylogenetic comparative methods [7]. Trees simulated under the same four diversification processes, plus a trait-dependent process [28], and mapped onto phylogenetic space (see Materials and Methods) corroborate that certain processes coincide with certain diversification types, but that the matching between type and process is not perfect. Trees generated under diversity-dependent and mass extinction models are the most localized in phylogenetic space, whereas other models are more widespread (S7 Fig). Nonetheless, the phylogenetic space of model-based trees largely corresponds (~85% overlap) with empirical phylogenetic space, and different regions of phylogenetic space are disproportionately associated with different diversification processes. Accordingly, these processes have shaped the geometry of vertebrate phylogenetic space by constraining family-level vertebrate phylogenies to diversify in particular ways.

**Fig 4:**
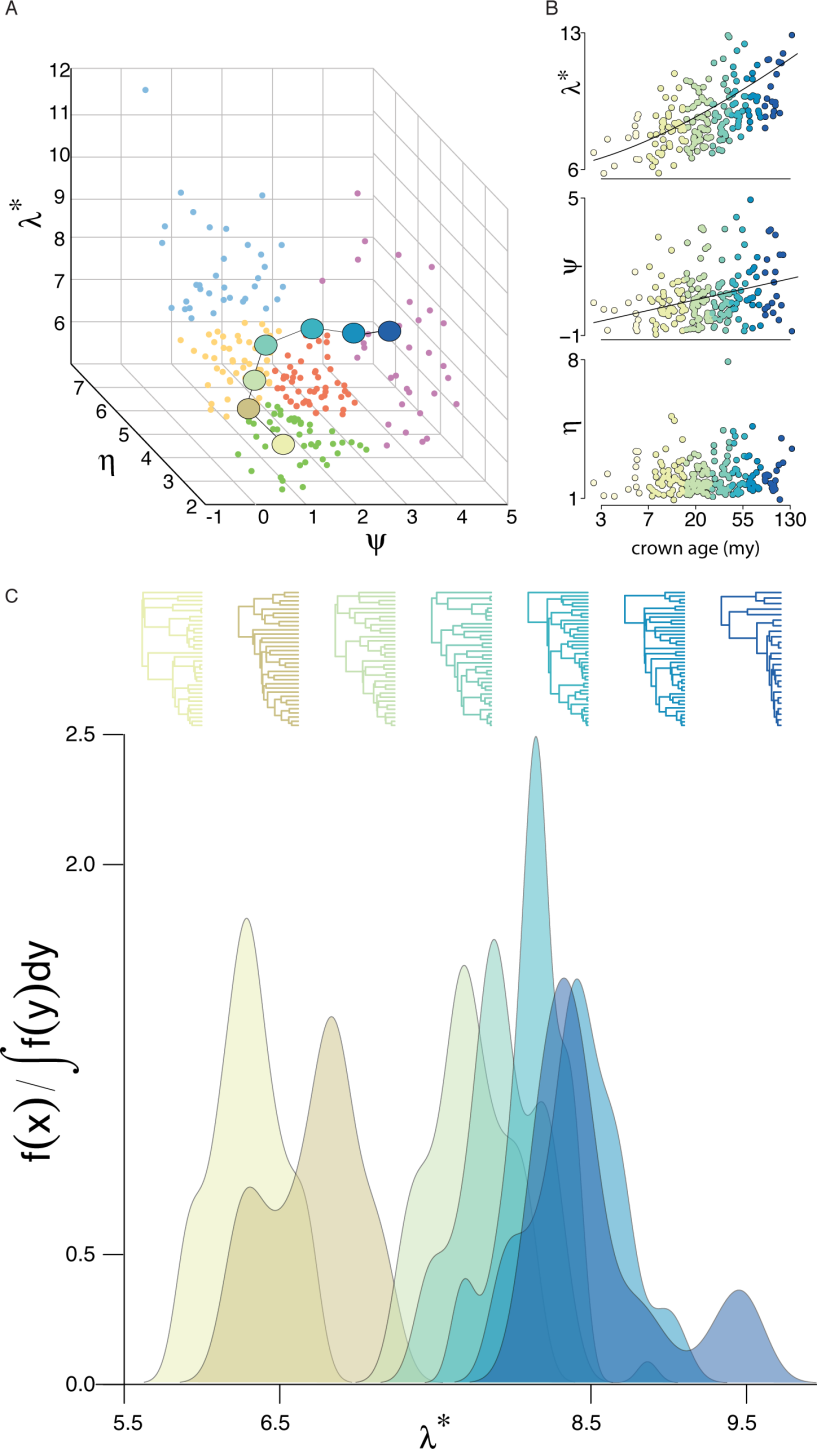
The average trajectory through phylogenetic space over time. All phylogenies were binned according to crown age. (A) The average position in phylogenetic space of each binned group. (B) The explicit relationship between crown age and λ* (*y* ∼ −0.42*x* + 0.41*x*^2^ − 0.02*x*^3^, *R*^2^ = 0.34, *p* < 0.001), *ψ* (*y* ∼ 0.6*x*, *R*^2^ = 0.15, *p* < 0.001), and *η* (*p* = 0.20). (C) Tree plots and spectral density profiles of the median phylogenies in each binned group.

We estimated the trajectory that, on average, families traverse through phylogenetic space over time by binning empirical phylogenies by crown age (see Materials and Methods). The trajectory traverses four diversification types starting from type II (the diversity-dependent type) followed by type I, III, and finally V (the mass extinction type) (Fig 4A). This is confirmed by a significant age dependence to each type (S8A Fig), although there is considerable variation within each bin (S8B Fig). The trajectory is characterized by an exponential increase in λ* and a linear increase in *ψ* (Fig 4B), but no significant trend in *η* or, despite its relevance to λ* [17], species richness (*p* = 0.34). However, when we look at taxonomic classes separately, we see that each takes a divergent average trajectory through phylogenetic space (S9 Fig), which conforms with their different distributions in space (S1B Fig) and suggests phylogenetic constraints to how diversification evolves. The trajectory for birds traverses a path involving types II/III, I, and V that resembles that of the global trajectory, whereas the mammalian trajectory takes a different path, traversing types II, III, and IV. It is notable that phylogenies that predate the Cretaceous–Paleogene (K–Pg) boundary (~66 million years [my]), which here comprise only non-mammalian families, fall within the region of phylogenetic space associated with mass extinction, whereas the most ancient mammalian phylogenies (<60 my) fall within the region associated with temperature dependence.

## Discussion

Finding general patterns in the way that clades diversify has been notoriously difficult, limiting our ability to understand how biodiversity emerges in deep time. Mapping clades in phylogenetic space allows us to have a much clearer view of the landscape of diversification. Importantly, we find that there are constraints to diversification. Vertebrate phylogenetic space fills only a third of all possible tree space—akin to the constrained morphospace of Raup‘s mollusc shells [29]—due to ostensible limitations to how vertebrate families successfully diversify. Biodiversity might well follow, if not entirely predictable, at least constrained trajectories through evolutionary time.

We find that there are five principal types of diversification, which are bound in phylogenetic space by three vertices characterized by: tippy trees; stemmy, irregular trees; and large, regular trees (vertices *a*, *b*, and *c* from Fig 2A, which correspond to type V, II, and IV trees, respectively). The bird family Acanthizidae is an example of archetype *a*; the mammalian family Muridae is an example of archetype *b*; and the mammalian family Nycteridae is an example of archetype *c*, while the mammalian family Leporidae is not optimized at any archetype but rather at an intermediate position in phylogenetic space. The support for mass extinction events among tippy trees suggests that the tippy part of phylogenetic space (high *ψ*) is occupied by clades that radiated after mass extinction left niche space unoccupied. The support for diversity-dependent models among disproportionally irregular stemmy trees (type II trees) suggests that the slowdown in speciation is differentially distributed across lineages due, perhaps, to differences in species abundances. Finally, support for temperature-dependent speciation among regular trees (type IV trees) suggests that radiation events resulting from environmental factors may similarly and pervasively affect all lineages. As certain regions of phylogenetic space are dominated by particular biotic or abiotic processes, it is ultimately the combined effect of intrinsic and extrinsic limiting factors on each family that shapes the geometry of phylogenetic space.

The three vertices bounding phylogenetic space are the result of trade-offs between various characteristics of the trees. There is a long history of work showing how phenotypic evolution is constrained in every instance: there are always trade-offs between optimizing different fitness strategies [30,31]. As a result, species adapt to one fitness optimum at the expense of others, or to a concessionary optimum that serves several while optimizing none [32]. While phylogenetic trees are not adaptive in the sense that phenotypes are, there are trade-offs and combinations of tree characteristics that are not seen in nature, suggesting that certain diversification patterns are unstable (i.e., they lead to clade-wide extinction). Trees simulated with a speciation rate > *α* * *e*^±0.1^, for example, fall overwhelmingly outside of vertebrate phylogenetic space. Therefore, successful diversification in vertebrate families is approximately bounded by these speciation rates, and the reason we see trade-offs in phylogenetic properties and a constrained phylogenetic space is because families that surpass those bounds go extinct. Consequently, we may expect phylogenies of extinct clades to violate these rules and fall outside of phylogenetic space.

Specifically, there are trade-offs between both stemminess and expansiveness with regularity among vertebrate families. Trees become more irregular as they become tippier. If tippyness is indeed the mark of mass extinction events, this trade-off could be the result of (mass) extinction unevenly culling parts of trees. We also observe a lower boundary for stemminess, which suggests that clades that diversify only early in their history go extinct. Finally, trees that are expansive tend to be more regular, suggesting that the various processes generating irregularity (e.g., runaway diversification in one lineage versus others, multiple slowdowns in diversification) result in contracted clades or widespread extinction.

As a consequence of these phylogenetic trade-offs, there are parts of phylogenetic space unoccupied by vertebrate families. Those parts are predominantly characterized by both stemmy and tippy regular trees and can be occupied by trees simulated with considerably decreasing and increasing rates of speciation, respectively. The fact that vertebrate family phylogenies are wedged between these two extremes of speciation provides the first evidence for the range of speciation possible at this level of vertebrate evolution. Such limitations to species diversification must be the consequence of a set of limiting factors such as minimum generation time, responsiveness to abiotic events, spatiotemporal availability of niche space, or some other facets of vertebrate speciation that favor certain diversification patterns while prohibiting others.

There is a significant positive correlation between crown age and both expansiveness and tippyness. The former correlation is unsurprising and suggests, firstly, that expansiveness is not bounded by any kind of upper ecological limit and, secondly, that branch length accumulates at a more or less similar rate across families, such that older families are typically more expansive than younger ones simply as a result of their having had more time to expand. The observation that tippyness also increases as a function of crown age is somewhat more surprising, although it is consistent with the observation that young clades tend to carry a stronger signal of early burst radiations (captured by stemminess) than older, more inclusive ones [9,19]. This suggests that young clades show a signal of early burst radiations, with speciation rates declining as ecological niches are filled, but also that this limitation in niche space is not absolute and radiations can happen later in a clade‘s history. As clades age, new opportunities for radiations may arise if, for example, ecological opportunity is recovered after a mass extinction event, or if new factors allow the exploitation of a part of ecological space that was not previously explored (e.g., new interspecific interactions prompting character displacement, fluctuations in temperature producing new niches under favorable environmental conditions for speciation).

The distinct distributions in and evolutionary trajectories through phylogenetic space of birds and mammals may reveal something about evolutionary differences between classes. Bird families are distributed nearly homogeneously in phylogenetic space and their average trajectory is similar to that of squamate, amphibian, and fish families. Mammals, on the other hand, are disproportionately underrepresented at archetype *a* and show an average trajectory through phylogenetic space orthogonal to those of other vertebrates. The relative preponderance of mammal families in diversification type IV is consistent with the metabolic theory of ecology [33] as well as work suggesting that mammalian diversification is inversely correlated with temperature [34]. We can speculate that the evolutionary novelty swaying mammalian biodiversity towards temperature dependency is connected to endothermic viviparity, which has led to considerable reproductive diversity in mammals [35] and has been shown to directly impact diversification in other vertebrates [36]. It is also worth noting that the clear age dependence of diversification types may bias the distribution of mammal families in phylogenetic space and, therefore, their relative absence near archetype *a* may be explained, at least in part, by their median youngness.

The benefit of generating an absolute phylogenetic space for species trees is that it provides a reference frame for describing and contextualizing phylogenies generally. It furthermore advances the idea that phylogenies are not simply the result of a single biotic or abiotic factor, but that a time-sensitive series of factors differentially influences diversification during a clade’s evolutionary history. By mapping non-vertebrate taxa in this space, it will be interesting to see whether the constraints observed here are unique to vertebrates or extend to other animal or even all organismal life.

## Materials and Methods

### Empirical Phylogenies

We compiled a dataset of dated, family-level ultrametric trees from an exhaustive literature search of species-level vertebrate phylogenies. In order to avoid biased comparisons between trees, we included only phylogenies that were ≥80% sampled, which is shown to be a sufficient sampling estimate to make use of the MGL [17]. We restricted our analyses to family-level trees because, after discarding insufficiently sampled trees, this provided us with the largest number of trees of adequate size (>20 species) while allowing us to cover all vertebrate classes. We used the most recent molecular mammalian tree with 4,160 extant species (v.1001) [18]. We used a molecular bird tree with 6,670 extant species constructed on the Hackett backbone [19]. To avoid known (and unknown) polytomies and negative branch-lengths in the mammal and bird phylogenies, we computed maximum clade credibility (MCC) trees in BEAST 2 [37] that selected trees with the highest posterior probability product. We used a phylogeny of 4,161 extant squamate species [20] and one of 3,309 extant amphibian species [21], both of which were constructed with a supermatrix analysis of molecular data. Finally, we used a phylogeny of 7,822 extant actinopterygians (ray-finned fishes), which was constructed with a maximum-likelihood approach and made ultrametric using penalized likelihood [22]. Our compilation resulted in 72 Mammalia, 102 Aves, 7 Squamata, 9 Amphibia, and 24 Actinopterygii family-level phylogenies for a total of 214 trees. To assess phylogenetic uncertainty, we randomly sampled 100 trees from posterior distributions of 1,000 mammal trees [18] and 10,000 bird trees (birdtree.org) and from each sample parsed family-level trees.

### Clustering of Trees Based on Spectral Density Profiles

Following the non-parametric approach described in detail in [17], given a phylogenetic tree, we defined its standard MGL as the difference between its degree matrix (the diagonal matrix where diagonal element *i* is the sum of the branch lengths from node *i* to all the other nodes in the phylogeny) and its distance matrix (where element (*i*,*j*) is the branch length between nodes *i* and *j*). We then obtained the spectral density profile of the tree by convolving the spectra of eigenvalues of the MGL with a Gaussian kernel. Finally, we measured the distance between phylogenies using the Jensen–Shannon index [38] and subjected these distances to unsupervised hierarchical and k-medoids clustering. For hierarchical clustering, significance was calculated with bootstrap resampling and clusters were considered significant at *α* ≥ 0.95. For k-medoids clustering, the number of clusters was estimated by optimum average silhouette width. Average silhouette widths greater than 0.5 were considered significantly supported. These analyses are implemented in the *R* package *RPANDA* [39].

To assess the effect of phylogenetic uncertainty on clustering results, we repeated both clustering protocols over 100 iterations by randomly drawing from the mammal and bird trees sampled from posterior distributions. We tallied the number of times we duplicated the original result using the MCC trees versus those where at least one tree was placed in a different cluster. When there were mismatches, we measured the percentage of trees that were placed in a different cluster.

We determined whether the distribution of trees across clusters was more or less than expected by chance for each taxonomic class. Given a specific taxonomic class represented by x trees, we iteratively distributed x hypothetical trees in the five clusters, where the probability of being placed in a particular cluster was defined by the relative size of that cluster (as calculated above). For each cluster, 500 iterations of this process yielded a null distribution of the expected number of trees in the cluster. We deemed significant the deviation of the actual number of families assigned to each cluster from the expected if the actual number fell in the lower or upper tail of the distribution (*α* < 0.05).

### Defining Phylogenetic Space

We computed the spectral density profile summary statistics (λ*, *ψ*, and *η*) of phylogenies as described in [17] (S1 Data). Briefly, each eigenvalue computed from the MGL of a phylogeny describes the distance between nodes, such that larger and smaller eigenvalues describe greater and shorter distances, which in practice represent speciation-poor and -rich regions of the tree, respectively. The largest distance, therefore, is denoted by the largest eigenvalue, λ*. When the spectrum of eigenvalues is convolved with a Gaussian kernel, it is possible to identify patterns in its distribution. The skewness, *ψ*, of the distribution indicates whether the eigenvalues of the MGL are preponderantly large or small and thus whether the phylogeny is comprised of mostly speciation-rich or -poor regions. Likewise, the peak height, *η*, demonstrates whether branch lengths in the phylogeny are heterogeneous (low *η*) or homogeneous (high *η*)—that is, irregular or regular, respectively. These statistics were used to map empirical phylogenies into a three-dimensional space. Spectral density profile summary statistics were regressed against each other and best-fit slopes were selected using a stepwise AIC. Modality in the MGL, which was not used to define phylogenetic space, was calculated using the eigengap heuristic (S1 Data) [17], which determines the number of modes of division in a phylogeny by the position of the greatest difference between ranked eigenvalues.

We determined the geometry of the space by finding the polytope with the smallest number of vertices (i.e., archetypes) that could explain the most variance (or beyond which little explanatory power was added) in the distribution of phylogenies [32]. Archetypes were positioned around the data using the SISAL algorithm and allowing for up to six archetypes. Best fits at *p* < 0.01 were determined by t-ratio tests. The phylogenetic features optimized at each archetype were determined by the relative contributions of each summary statistic at that archetype [23]. To assess how sufficiently our sample of phylogenies captured the entirety of vertebrate phylogenetic space, we performed rarefaction analyses. We calculated the maximum coordinates of the archetypes for bootstrapped samples of phylogenies while fixing the number of dimensions at three and fit saturation curves for those coordinates as a function of sample size. Bootstrapped samples were replicated 50 times.

### Characterizing the Phylogenetic Space Available to Trees

To assess the entirety of phylogenetic space available to trees consistent with the empirical distribution of λ*, we extensively generated phylogeny-type networks (i.e., bifurcating, ultrametric, rooted networks) while constraining λ* to be in the empirical distribution. Given a λ* from the empirical distribution, we constructed a graph Laplacian by populating a symmetric, positive, semidefinite M-matrix with zero-sum rows and columns. We computed a distance matrix from the graph Laplacian using the classical Dijkstra algorithm, which finds distances between nodes in a graph given an adjacency matrix [40]. We obtained the corresponding non-ultrametric tree from the distance matrix using complete linkage clustering [41], and the resulting tree was made ultrametric using mean path length [42]. Finally, the tree was rooted at the stem and, where necessary, polytomies were resolved using the *R* package *ape* [43]. We constructed 10,000 such trees. The proportion of phylogenetic space available to trees that is occupied by empirical phylogenies was calculated as the volume of three-dimensional space occupied between the most distal empirical points along each axis as a percentage of the total space occupied by the simulated trees. The geometry of tree-type space was determined with a polytope analysis as above, using 1,000 trees randomly sampled from the 10,000 for computing purposes.

To interpret phylogenetic space in terms of statistical diversification processes, we simulated ultrametric birth–death trees using the *R* package *TESS* [44]. We simulated 500 trees at ages 10–60 my under constant speciation rates (b = 0.1–0.25), decreasing speciation rates (*α* * *e*^−*βt*^ for *α* = 0.01–−1 and *β* = 0.05–−0.2, and increasing speciation rates (*α* * *e*^*βt*^ for *α* = 0.01–−0.05 and *β* = 0.05–−0.2); the extinction rate was held constant (d = 0–0.1). We discarded trees outside the range of species richness for empirical phylogenies (20–700). The final set of trees for each birth–death model had a comparable mean species richness to the empirical set. We then computed their spectral density profiles and plotted them in phylogenetic space as above.

### Interpreting Phylogenetic Space in Macroevolutionary Terms

We selected phylogenetic diversification models meant to directly model a process (rather than a temporal trend) and that were comparable in a likelihood framework. This resulted in five models featuring mass extinction events, linear and exponential diversity dependence of speciation rates, exponential temperature dependence, and SGD. The mass extinction model was fitted using the |bd.shifts.optim| function in the *R* package *TreePar*, which maximizes the likelihood of a model with constant speciation and extinction rates and one or more sampling events (i.e., mass extinctions) at discrete time points *t*_1_,*t*_2_,…,*t*_*n*_ in the history of the clade [24]. We allowed the mass extinction events to occur at any time. Diversity-dependent models were fitted using the dd_ML function in *DDD* [25]. We tested models with speciation rates showing both linear and exponential dependence to the number of extant species. Temperature-dependent models were fitted with the fit_env function in *RPANDA* with constant, linear, and exponential speciation—and extinction-rate dependence to temperature [26]. Temperature data were inferred from benthic foraminiferal *δ*^18^*O* measurements extending to 108 my ago [45] at 0.1 my time-steps. Finally, the SGD model was fit using the fit_sgd function in *RPANDA* with Nelder–Mead optimization [27]. We fitted these models to all empirical phylogenies with ≥50 species (a total of 62 phylogenies). Likelihood values were normalized after [46] in order to make the likelihood estimates directly comparable. Models with the lowest AICc scores were considered the most supportive of the phylogeny [47]. Trait-dependent models were not considered, as their likelihood functions are not, in their current form, comparable to the likelihoods of the other models.

We simulated trees under five diversification models—birth–death with mass extinction, exponential diversity dependence (which was always favored to linear dependence), exponential temperature dependence, SGD, and continuous trait dependence [28]—and mapped those trees in phylogenetic space. In order to restrict the parameter space to realistic values, we first estimated parameters by fitting models to the 62 empirical phylogenies with ≥50 species as above. We fitted the continuous trait-dependent model with ln-transformed body mass (or, for ray-finned fish, maximum length) as trait data using QuaSSE in *diversitree* [48]. Data were collected from the literature for mammals [49], birds [50], and ray-finned fish [51]. For each empirical parameter estimate, 30 trees were simulated, resulting in 1,500 simulated trees per diversification model. We calculated the distribution of simulated trees in phylogenetic space by drawing polygons around each empirical diversification type and counting the number of trees simulated under a diversification model falling in each polygon. Polygon vertices were selected using the most distal points in each type. The volume of phylogenetic space for simulated trees was estimated as above and the percentage correspondence of space between empirical and simulated trees was calculated as the volume of overlapping space over the total volume occupied by both empirical and simulated trees.

### Drawing Evolutionary Trajectories through Phylogenetic Space

We binned empirical phylogenies by crown age using a natural breaks optimization algorithm [52] without specifying a number of desired bins. For each bin, we calculated the arithmetic mean for λ*, *ψ*, and *η* and then plotted those values in empirical phylogenetic space. The diversification type corresponding to each bin was determined by drawing polygons around each diversification type as above. Median phylogenies (Fig 4C) were defined as those falling nearest the arithmetic means for λ*, *ψ*, and *η*. The same was done for mammals and birds separately. There were too few trees in the other taxonomic classes to analyze them similarly.

## Supporting Information

S1 Data Statistics for vertebrate phylogenies. (CSV)

S2 Data Distribution of diversification types across classes. (CSV)

S3 Data Deviation of classes across diversification types from null distribution. (CSV)

S4 Data Percentage of support for each model among empirical trees in different regions of phylogenetic space. (CSV)

S5 Data Spectral density profile summary statistics for trees simulated under different models. (CSV)

S6 Data Percentage of models falling within each region of phylogenetic space. (CSV)

S1 Fig (A) The deviation of the number of families of each diversification type in each class (see Fig 1E) from an expected null distribution based on the number of families in each class and in each diversification type. Asterisks denote a significant deviation at *p* < 0.05. (B) Boxplot of mean crown age for each phylogenetic class (S1 Data). Phylogenies sampled for squamates, amphibians, and fish are significantly (T > 2.51, *p* < 0.05) older than those sampled for birds and mammals. (C) Plots of the distribution of mammals (top), birds (middle), and other vertebrate families (bottom) in phylogenetic space. See S1 Data and S3 Data.
(TIF)

S2 Fig The distribution of sampling fractions in phylogenetic space. (A) Empirical phylogenies in phylogenetic space, colored according to the sampling fraction available for that phylogeny (S1 Data): 100% (black), 90%–99% (red), and 80%–89% (green). (B) Histograms of λ* (top), *η* (middle), and *ψ* (bottom) across trees of a given sampling fraction. Differences between the distributions of trees of different sampling fractions, calculated by a Kolmogorov-Smirnov test, are not significant along either axis (*D* > 0.15, *p* > 0.42).
(TIF)

S3 Fig The geometry of tree-type space computed on spectral density summary statistics for 1,000 simulated MGLs (randomly sampled from 10,000 simulated). The distribution cannot be entirely encompassed by a polytope; variability is best explained by a single point.
(TIF)

S4 Fig Max coordinates for each vertex in 2-D triangular phylogenetic space (see Fig 2B) for bootstrapped samples of empirical phylogenies. Error bars represent standard deviations calculated from 50 replicates. Logarithmic curves were fit to data for each vertex (*p* < 0.01).
(TIF)

S5 Fig (A) Ultrametric trees simulated under decreasing rates of speciation (0.8 * *e*^−0.1*t*^; red) and increasing rates of speciation (0.04 * *e*^0.1*t*^; green) projected into empirical phylogenetic space. Constant-rate trees (not shown) fell within the range of empirical trees. (B) Trade-offs between phylogenetic properties in phylogenetic space. Statistics for best-supported relationships for decreasing speciation rate trees are shown in red on the right. No significant (*p* < 0.05) relationships were found between phylogenetic properties for increasing speciation-rate trees.
(TIF)

S6 Fig Boxplot of the ΔAICc between models fit to empirical phylogenies (see Fig 2C). For all phylogenies best supported by a certain model, we computed the ΔAICc as the difference between the best and second-best supported model for each phylogeny. See S1 Data.
(TIF)

S7 Fig The percentage of trees simulated under different diversification models that fall within the phylogenetic space occupied by each empirical diversification type (numerals refer to types). See S5 Data and S6 Data.
(TIF)

S8 Fig (A) Boxplot of mean crown age for each diversification type. (B) Boxplot of mean λ*, *ψ*, and *η* for each binned age group. See S1 Data.
(TIF)

S9 Fig The average trajectory of families from different classes through phylogenetic space over time. (A) Families from mammals and birds were separately binned into seven bins by crown age based on Jenks optimization. A line is drawn through the average position in phylogenetic space for each binned group. (B) The best-fit regression slope for λ* (mammals, *y* ∼ −0.644*x* + 0.338*x*^2^, *R*^2^ = 0.32, *p* < 0.01; birds, *y* ∼ 0.243*x*, *R*^2^ = 0.23, *p* < 0.01), *ψ* (mammals, *R*^2^ = 0.03, *p* = 0.07; birds, *y* ∼ 0.10*x*, *R*^2^ = 0.05, *p* < 0.015), and *η* (mammals, *y* ∼ 0.108*x*, *R*^2^ = 0.05, *p* = 0.03; birds, *R*^2^ = −0.01, *p* = 0.94) as a function of crown age for each clade (S1 Data). (C) Representative spectral density profiles and trees for each binned group in mammals (top) and birds (bottom). Boxes around trees are colored according to the diversification type that bin coincides with in phylogenetic space.
(TIF)

## Abbreviations

AICc: Akaike Information Criterion
MCC: maximum clade credibility
MGL: modified graph Laplacian; my, million years
SGD: speciation by genetic differentiation
