# Supplementary figures and images for: Natural Constraints to Species Diversification
Source: PLoS Biol. 2016 Aug 9;14(8):e1002532. doi: 10.1371/journal.pbio.1002532 (PMC4978419; doi:10.1371/journal.pbio.1002532)

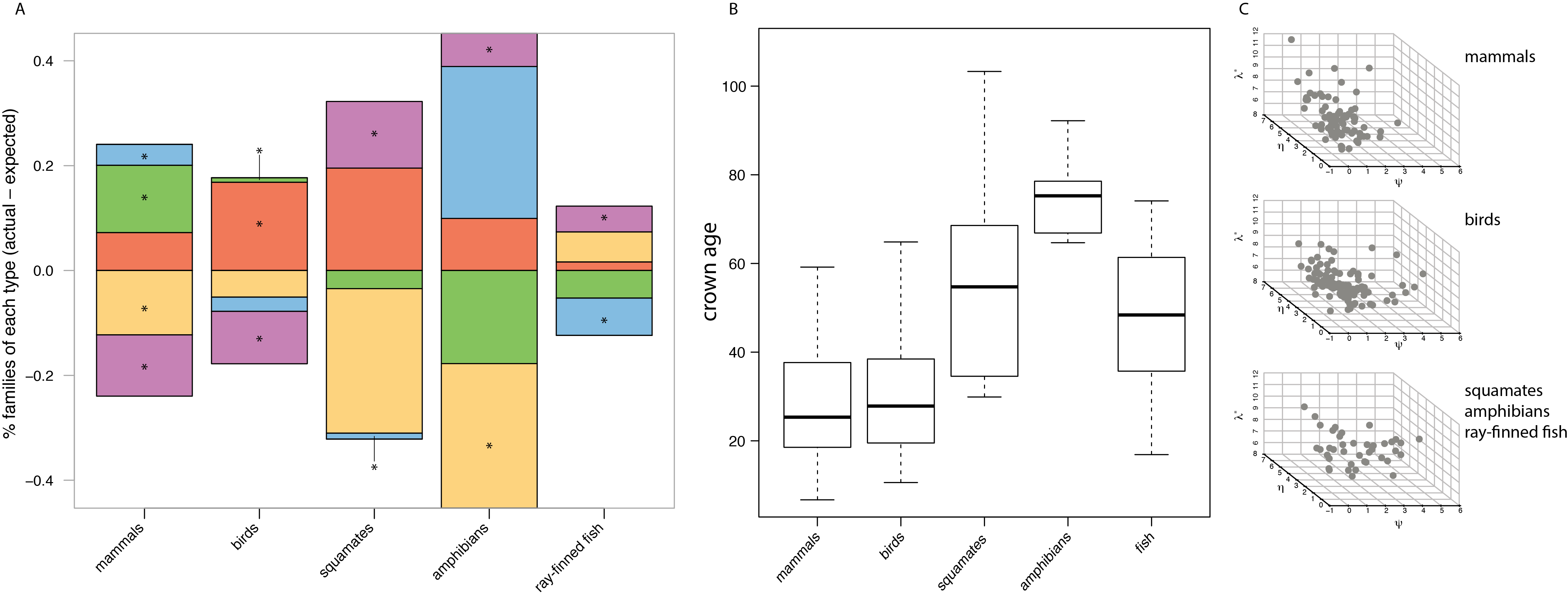

Supplement: S1 Fig — (A) The deviation of the number of families of each diversification type in each class (see Fig 1E) from an expected null distribution based on the number of families in each class and in each diversification type. Asterisks denote a significant deviation at p < 0.05. (B) Boxplot of mean crown age for each phylogenetic class (S1 Data). Phylogenies sampled for squamates, amphibians, and fish are significantly (T > 2.51, p < 0.05) older than those sampled for birds and mammals. (C) Plots of the distribution of mammals (top), birds (middle), and other vertebrate families (bottom) in phylogenetic space. See S1 Data and S3 Data. (TIF) [file pbio.1002532.s007.tif]

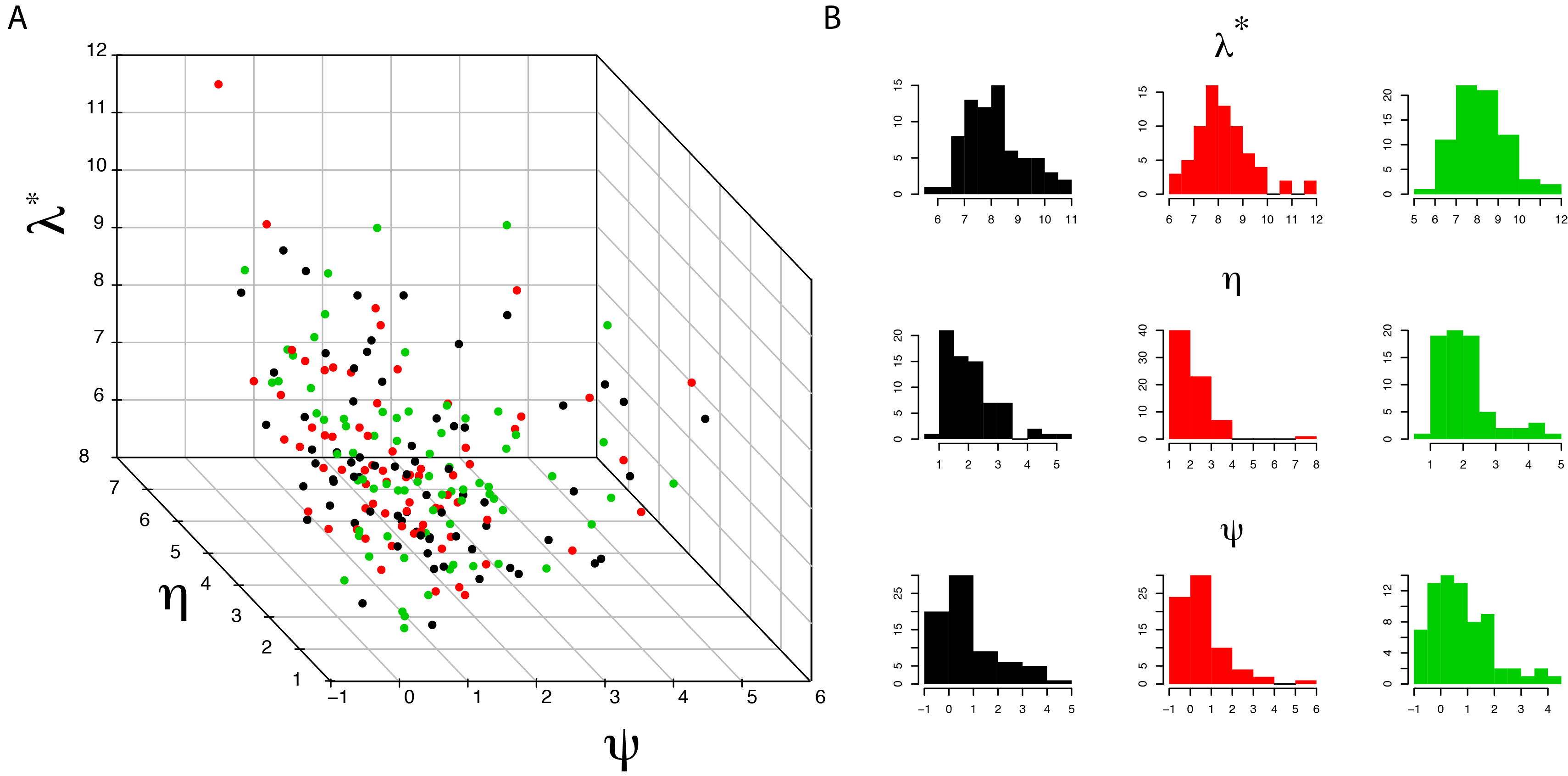

Supplement: S2 Fig — (A) Empirical phylogenies in phylogenetic space, colored according to the sampling fraction available for that phylogeny (S1 Data): 100% (black), 90%–99% (red), and 80%–89% (green). (B) Histograms of λ* (top), η (middle), and ψ (bottom) across trees of a given sampling fraction. Differences between the distributions of trees of different sampling fractions, calculated by a Kolmogorov-Smirnov test, are not significant along either axis (D > 0.15, p > 0.42). (TIF) [file pbio.1002532.s008.tif]

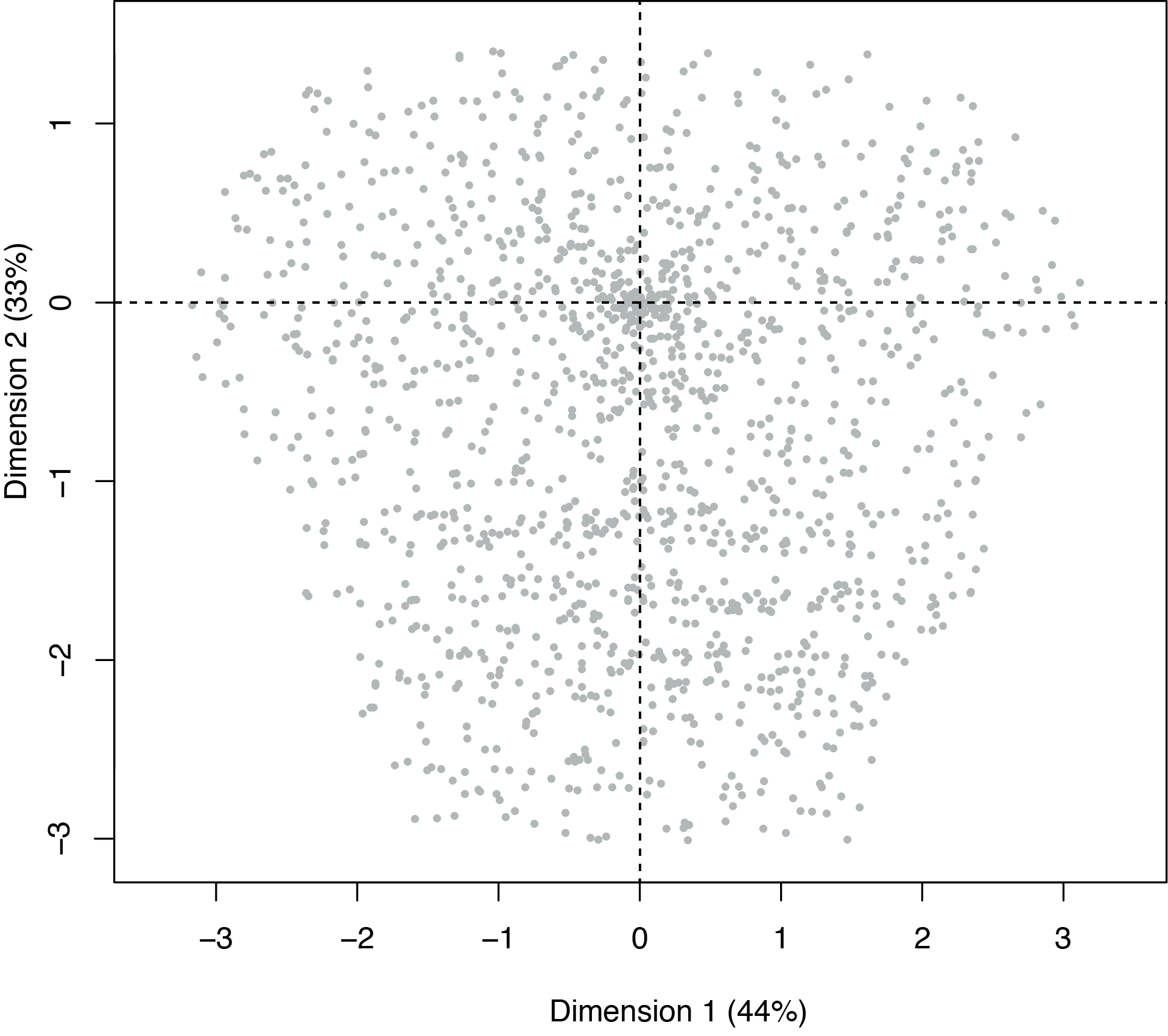

Supplement: S3 Fig — The distribution cannot be entirely encompassed by a polytope; variability is best explained by a single point. (TIF) [file pbio.1002532.s009.tif]

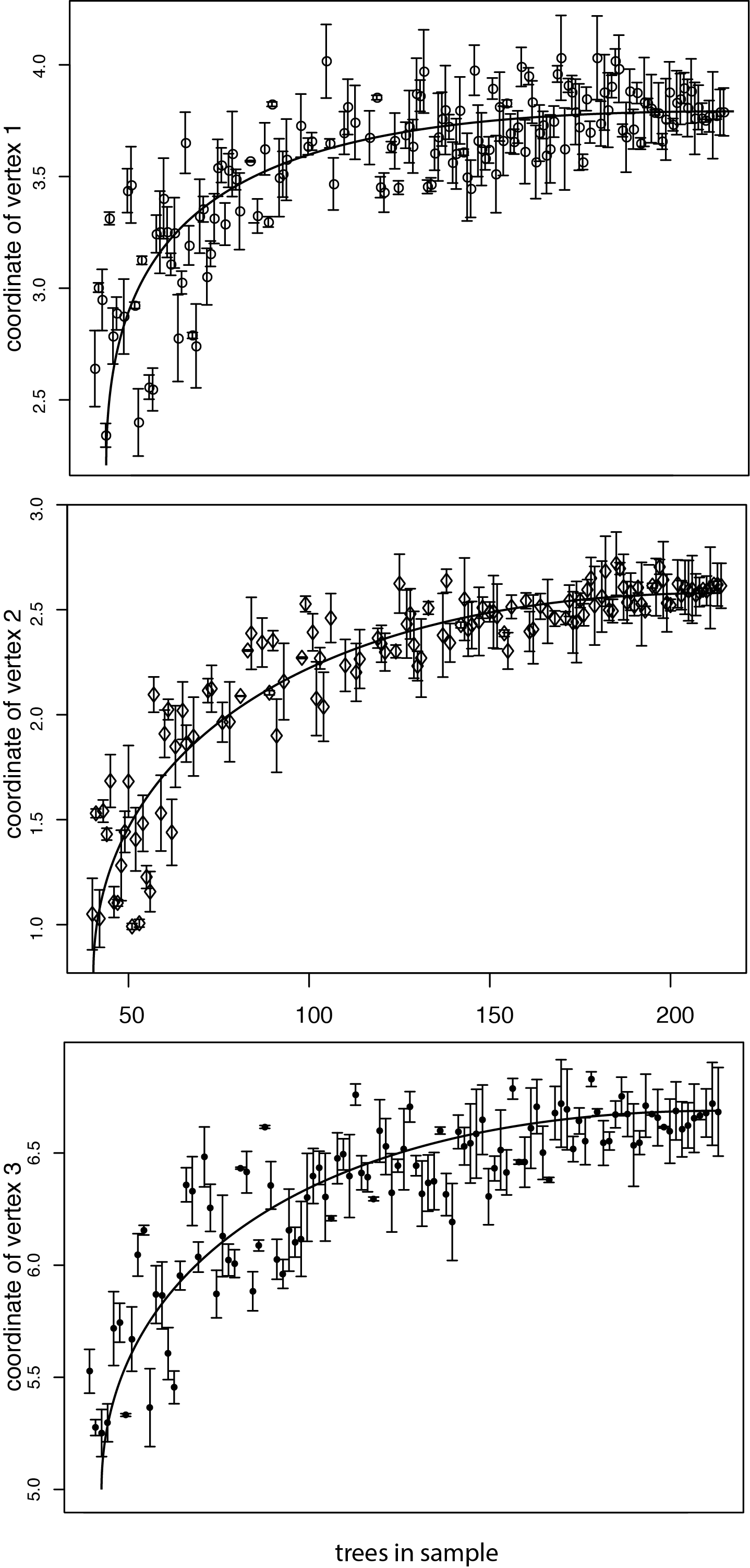

Supplement: S4 Fig — Error bars represent standard deviations calculated from 50 replicates. Logarithmic curves were fit to data for each vertex (p < 0.01). (TIF) [file pbio.1002532.s010.tif]

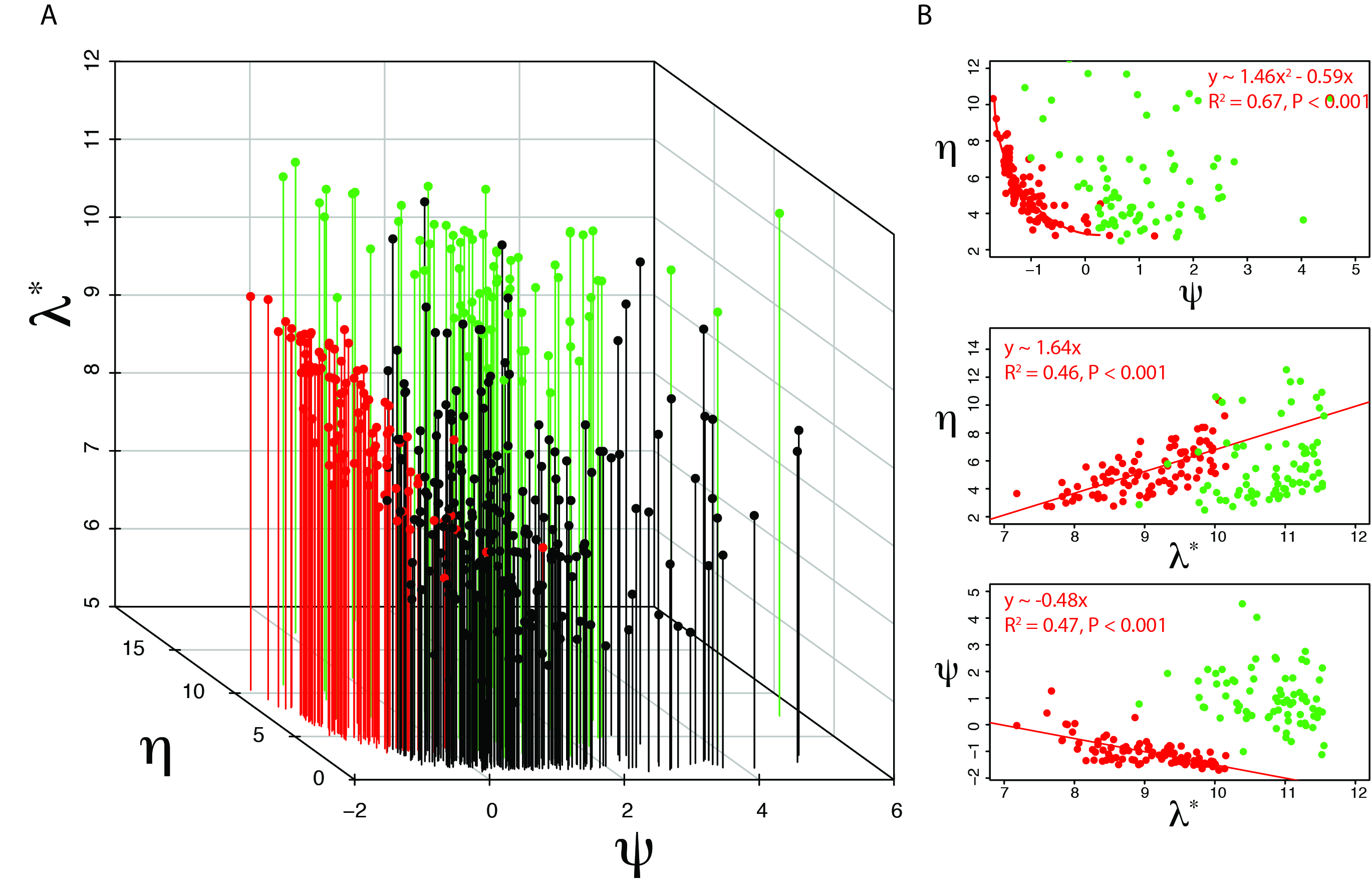

Supplement: S5 Fig — (A) Ultrametric trees simulated under decreasing rates of speciation (0.8 * e−0.1t; red) and increasing rates of speciation (0.04 * e0.1t; green) projected into empirical phylogenetic space. Constant-rate trees (not shown) fell within the range of empirical trees. (B) Trade-offs between phylogenetic properties in phylogenetic space. Statistics for best-supported relationships for decreasing speciation rate trees are shown in red on the right. No significant (p < 0.05) relationships were found between phylogenetic properties for increasing speciation-rate trees. (TIF) [file pbio.1002532.s011.tif]

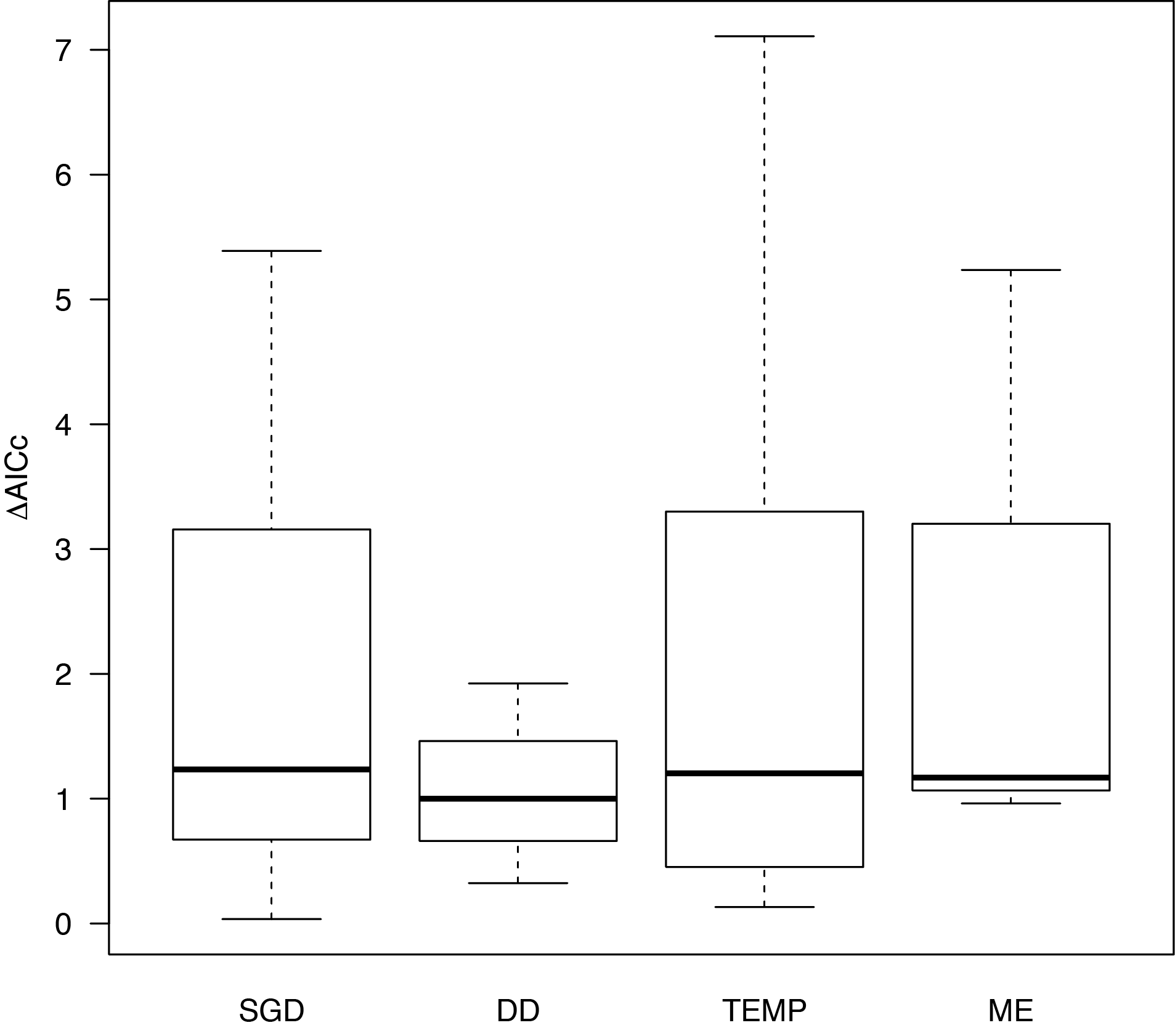

Supplement: S6 Fig — For all phylogenies best supported by a certain model, we computed the ΔAICc as the difference between the best and second-best supported model for each phylogeny. See S1 Data. (TIF) [file pbio.1002532.s012.tif]

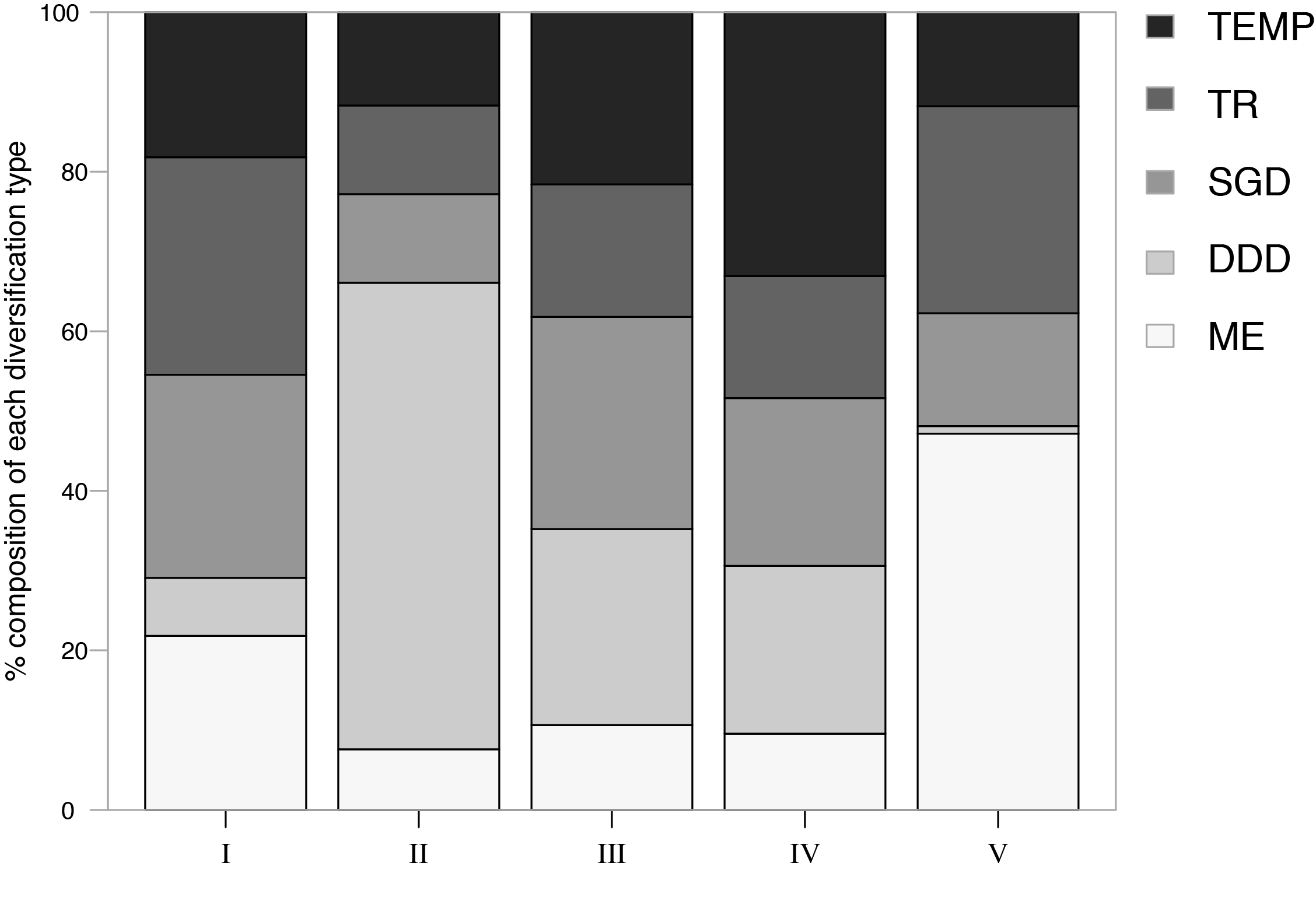

Supplement: S7 Fig — See S5 Data and S6 Data. (TIF) [file pbio.1002532.s013.tif]

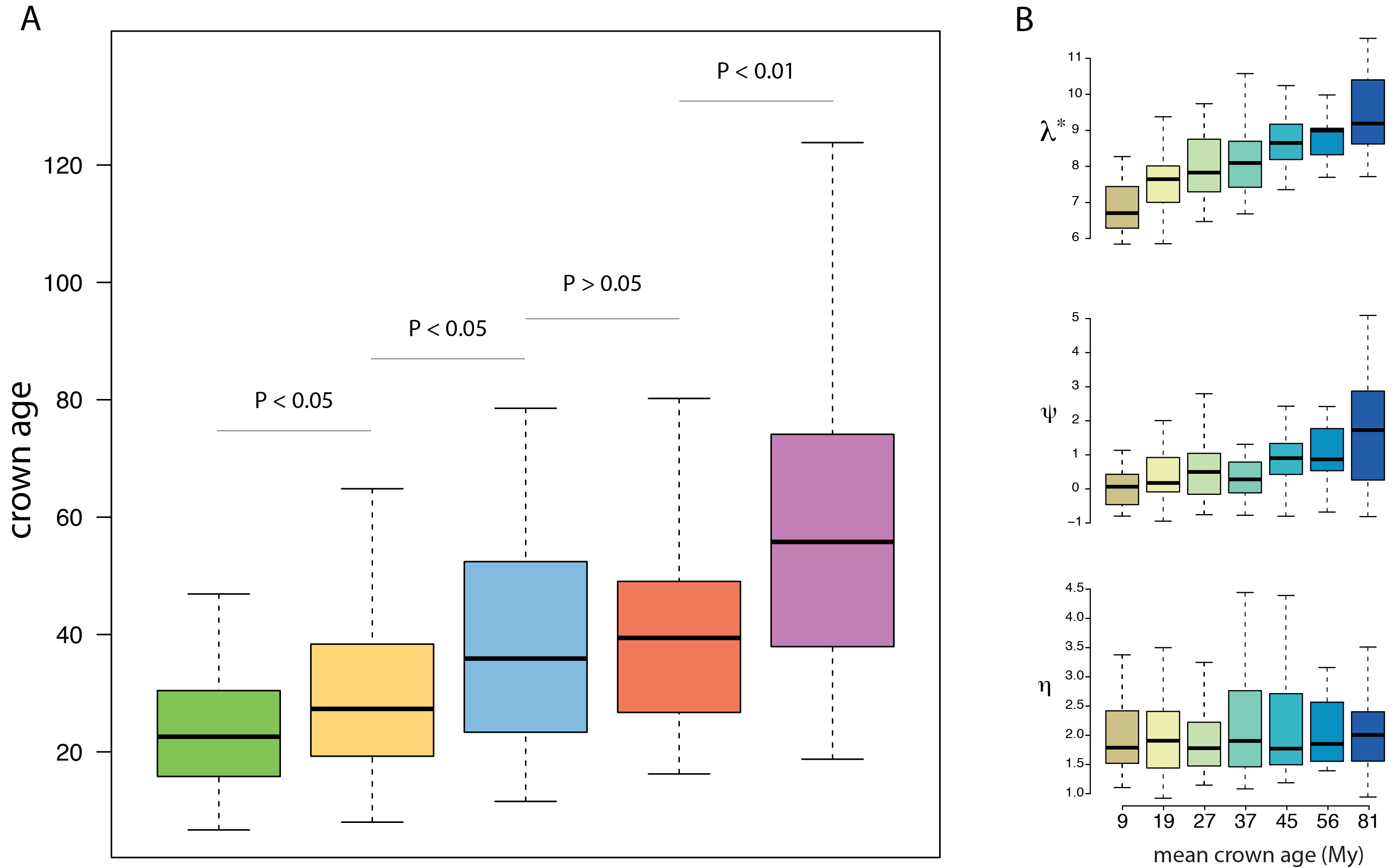

Supplement: S8 Fig — (A) Boxplot of mean crown age for each diversification type. (B) Boxplot of mean λ*, ψ, and η for each binned age group. See S1 Data. (TIF) [file pbio.1002532.s014.tif]

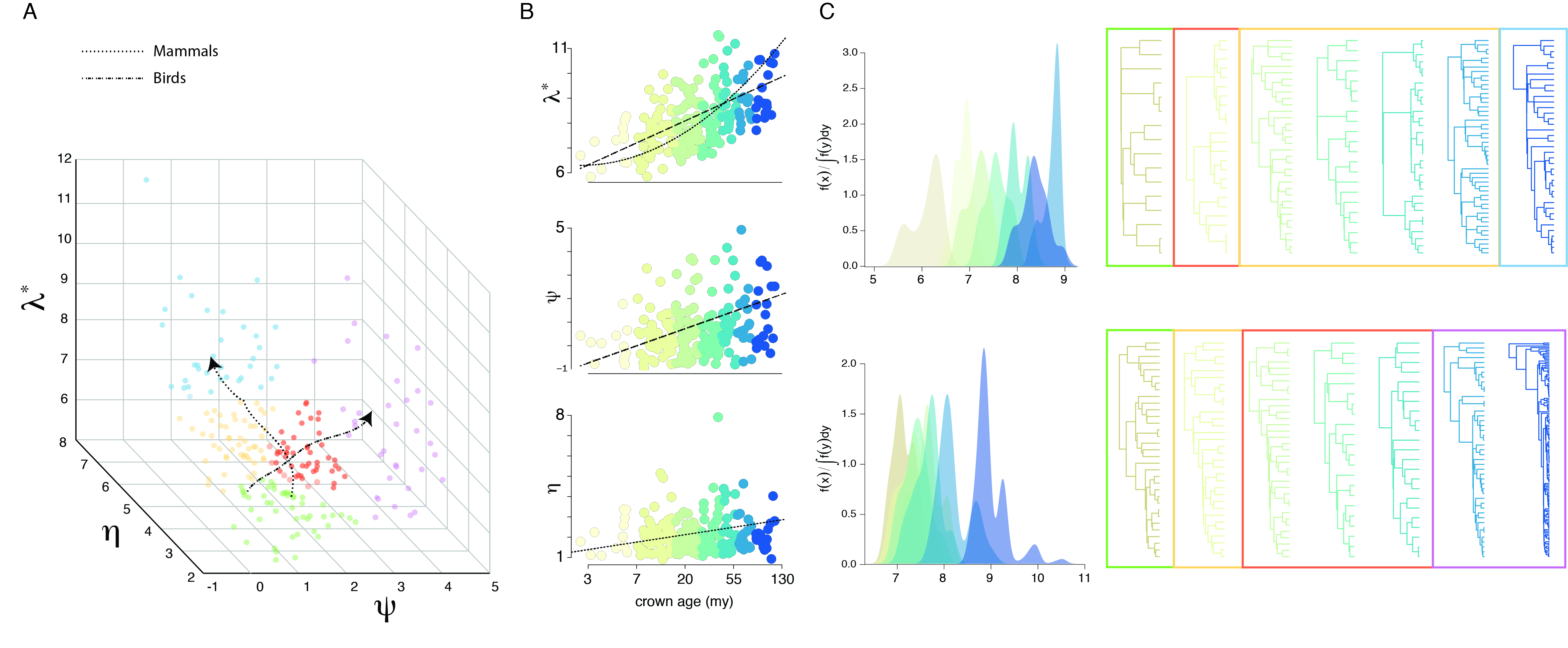

Supplement: S9 Fig — (A) Families from mammals and birds were separately binned into seven bins by crown age based on Jenks optimization. A line is drawn through the average position in phylogenetic space for each binned group. (B) The best-fit regression slope for λ* (mammals, y ∼ −0.644x + 0.338x2, R2 = 0.32, p < 0.01; birds, y ∼ 0.243x, R2 = 0.23, p < 0.01), ψ (mammals, R2 = 0.03, p = 0.07; birds, y ∼ 0.10x, R2 = 0.05, p < 0.015), and η (mammals, y ∼ 0.108x, R2 = 0.05, p = 0.03; birds, R2 = −0.01, p = 0.94) as a function of crown age for each clade (S1 Data). (C) Representative spectral density profiles and trees for each binned group in mammals (top) and birds (bottom). Boxes around trees are colored according to the diversification type that bin coincides with in phylogenetic space. (TIF) [file pbio.1002532.s015.tif]
